# miR-16-5p Suppresses Progression and Invasion of Osteosarcoma *via* Targeting at Smad3

**DOI:** 10.3389/fphar.2020.01324

**Published:** 2020-08-26

**Authors:** Zhijian Gu, Zhikun Li, Ruijun Xu, Xiaodong Zhu, Ruixi Hu, Yonghua Xue, Wei Xu

**Affiliations:** ^1^ Department of Orthopedics, Tongren Hospital, School of Medicine, Shanghai Jiao Tong University, Shanghai, China; ^2^ Department of Neurosurgery, Putuo District Central Hospital, Shanghai University of Traditional Chinese Medicine, Shanghai, China

**Keywords:** osteosarcoma, miR-16-5p, invasion, Smad3, proliferation

## Abstract

**Background:**

MicroRNAs are known to regulate carcinogenesis of osteosarcoma. Although, miR-16-5p is known to exert inhibitory effects on several forms of cancers, its effects on the growth and invasion of osteosarcoma have not been studied.

**Methods:**

We collected human osteosarcoma specimens and adjacent tissues to detect the expression of miR-16-5p by real-time polymerase chain reaction, immunoblotting, and immunohistochemistry. The proliferation, migration, and invasion of MG63 and HOS cells following miR-16-5p overexpression and inhibition were detected with cell counting kit-8, wound healing assay, and Transwell assay, respectively. An expression vector carrying a mutated 3′-untranslated region of mothers against decapentaplegic homolog 3 (Smad3) was constructed.

**Results:**

The results showed that miR-16-5p expression was downregulated in osteosarcoma tissues and cells as compared with adjacent counterparts, while Smad3 was overexpressed in osteosarcoma cells. The overexpression of miR-16-5p resulted in the inhibition of the proliferation, migration, and invasion of osteosarcoma cells and enhanced the therapeutic effect of cisplatin. These effects were attenuated with miR-16-5p expression inhibition. In cells transfected with miR-16-5p mimic, Smad3 expression decreased, while this effect was absent in the cells carrying mutated Smad3.

**Conclusions:**

Therefore, miR-16-5p inhibits the growth and invasion of osteosarcoma by targeting Smad3.

## Highlights

This study firstly investigated that miR-16-5p inhibited the proliferation, migration, and invasion of osteosarcoma *via* suppressing the expression of Smad3, meaning that miR-16-5p may be regarded as a potential target in the treatment of osteosarcoma.

## Introduction

Osteosarcoma is regarded as the most common primary malignant mesenchymal tumor of bones in both adults and adolescents (Yang and Zhang, 2013). Although many novel anti-cancer drugs or molecular targeted therapies have been developed over last few decades, the 5-year survival rate of patients with osteosarcoma is still around 60% (Ritter and Bielack, 2010). However, the molecular mechanisms underlying carcinogenesis of osteosarcoma remain unknown and may involve mutations at genomic, transcriptomic, and proteomic levels (Scotlandi et al., 1996).

Epigenetic events can cause tumorigenesis (Foley et al., 2015; Chano et al., 2016; Li et al., 2018). As small non-coding RNAs, micro-RNAs (miRNAs) are short single-stranded ribonucleic acid (usually contain 17–25 nucleotides) that may complementarily pair with the 3′-untranslated regions (3′-UTRs) of their target messenger RNAs (mRNAs) and induce gene-silencing effects through the degradation of their targets. Studies have demonstrated that miRNA may modulate the chemotherapeutic sensitivity of osteosarcoma cells (Meng et al., 2017; Song et al., 2017; Wang et al., 2017). miR-16-5p has been demonstrated as an vital component of the intracellular micro-RNA regulatory network. A remarkably inhibitory effect of miR-16-5p on the growth and invasion of cancer, including breast cancer, gastric cancer, osteosarcoma, and chordoma, has been reported previously (Rinnerthaler et al., 2016; Qu et al., 2017; Sang et al., 2017; Zhu et al., 2018; Zhang et al., 2018). Exosome-associated micro-RNA panels were recently thought to predict drug resistance in patients with multiple myeloma, wherein the expression of miR-16-5p was found to be downregulated in patients resistant to bortezomib (Zhang et al., 2016). miR-16-5p is shown to inhibit gastric cancer, breast cancer, and chordoma in previous researches (Zhang et al., 2015; Rinnerthaler et al., 2016; Zhu et al., 2018; Zhang et al., 2018). However, the function of miR-16-5p in the progression and chemosensitivity of osteosarcoma still remains unknown.

Smad3, as an important transporter in the transforming growth factor β (TGF-β) signaling pathway, can transports the TGF-β signal from the cell membrane into the nucleus, so that the TGF-β can combine with relevant nuclear factors to regulate the expression of target genes, thereby, for controlling cell proliferation. Abnormal expression and function of smad3 will lead to dysfunction of proliferation, differentiation, migration, and apoptosis of cell, and eventually can trigger the occurrence, progression, and metastasis of cancer (Tang et al., 2017; Rypens et al., 2020). It was revealed that osteosarcoma cells could release TGF-β, which can be suppressed by inhibitors of TGF-β type I receptor and knockdown of Smad3. Moreover, significant expressions of CD42b, TF, TGF-β, Smad2/3, and p-Smad2/3 were also detected in a biopsy sample from an osteosarcoma patient compared with control. Collectively, these finding suggested that the interaction between osteosarcoma cells and platelets, *via* thrombin and TGF-β, results in a continuous cycle, and that anti-TGF-β or anti-Smad3 therapy could be a promising tool for disease treatment (Saito et al., 2018).

In the present study, we reveal the significant decrease in the expression level of miR-16-5p in osteosarcoma cells as compared with normal cells. Upregulation in miR-16-5p expression significantly inhibits the proliferation, migration, and invasion of osteosarcoma cells by silencing mothers against decapentaplegic homolog 3 (Smad3) expression.

## Methods and Materials

### Tissue Samples of Patients and Cell Culture

The studies involving human participants were reviewed and approved by Institutional Review Board of Tongren Hospital (No. 2019-015). The patients provided their written informed consent to participate in this study. The informed consent in accordance with the Declaration of Helsinki were signed and obtained from all donors.

In this study, osteosarcoma specimens were obtained from 40 patients that underwent surgical resections in Orthopedics of Tongren Hospital from June 2012 to October 2014. The tissues were snap-frozen in liquid nitrogen and stored at −80°C. Adjacent soft tissues were obtained from the same patients. The clinical and pathological materials of patients were displayed in **Table 1**. A human osteoblast precursor cell line, hFOB1.19, and osteosarcoma cell lines, MG63, SaOS2, HOS, and U2OS, were provided by the American Type Culture Collection (ATCC). Dulbecco’s modified Eagle’s medium (DMEM) added with 10% fetal bovine serum (FBS) were used to culture cells in an atmosphere at 5% CO_2_ and 37°C.

**Table 1:** Clinical-pathological materials of patients with osteosarcoma.

**Clinical and pathological parameters**	**Total (n)**	**miR-16-5p expression**		
		High	Low	*p*-value
**Total**	40	23	17	
**Gender**				0.5473 (*NS*)
Male	21	13	8	
Female	19	10	9	
**Age, years**				0.6798 (*NS*)
** **<50	15	8	7	
** **>50	25	15	10	
**Histological stage**				0.049*
** **Well differentiated	19	14	5	
** **Poor differentiated	21	9	12	
**Tumor diameter, cm**				0.001*
** ≤5**	19	16	4	
** ＞5**	29	7	13	
**TNM stage**				0.002*
** **I and II	23	18	5	
** **III and IV	17	5	12	

NS, no significant.

*means p < 0.05.

### Cell Transfection

The siRNA targeting Smad3, scrambled negative control (NC) siRNA, miR-16-5p mimic, miR-NC, miR-16-5p inhibitor, and NC inhibitor were obtained from GenePharma (Suzhou, Jiangsu, China). MG63 and HOS cells were transfected with siRNA, miR-16-5p mimic, miR-NC, miR-16-5p inhibitor, and NC inhibitor using Lipofectamine 3000 (Invitrogen), as per the manufacturer’s instructions. The final concentrations of miRNA mimic and siRNA were 100 and 20 nM, respectively.

The sequences were as follows:

miR-16-5p mimic: 5′-UAGCAGCACGUAAAUAUUGGCG-3′

miR-16-5p inhibitor: 5′-CACCAAUAUUUACGUGCUGCUA-3′

miR-NC: 5′-UUCUCCGAACGUGUCACGUTT-3′

Smad3 siRNA: 5′-CCGCAU GAGCUUCGUCAAATT-3′

### Cell Viability Assay

The proliferation of cells was evaluated using cell-counting kit-8 (CCK-8). Briefly, HOS or MG63 cells transfected with miR-16-5p mimic, miR-NC, miR-16-5p inhibitor, or NC inhibitor were seeded in 96-well plates at 1 × 10^4^ cells/well and incubated for 24, 48, 72, and 96 h. At each time point, the media were discarded and replaced with fresh serum-free media containing CCK-8 solution at a final concentration of 10% (v/v). The plates were incubated in the dark at 37°C and 5% CO_2_ atmosphere for 2 h. Then, the ODs were measured using a microplate reader at 450 nm. All results were obtained from three independent experiments.

### Total Messenger RNA Extraction and Real-Time Polymerase Chain Reaction (RT-PCR) Assay

Total mRNAs were extracted using the Qiagen RNeasy^®^ Mini Kit (Valencia, CA, USA) according to the instructions provided by the manufacturer before reverse transcription into complementary DNA (cDNA). Real-time PCR (RT-PCR) was conducted with SYBR^®^ Premix Ex TaqTM Kit on Applied Biosystems 7500 Real-Time PCR instrument. The U6 small nuclear RNA and glyceraldehyde-3-phosphate dehydrogenase (*GAPDH*) mRNA were used as internal controls for miR-16-5p and *Smad3* mRNA, respectively. The sequences of primers for RT-PCR were as follows:

Smad3 forward primer,  5′-GTCTGCAAGATCCCACCAG-3′ and

reverse primer,  5′-AGCCCTGGTTGACCGACT-3′;

GAPDH forward primer,  5′-ACCCAGAAGACTGTGGATGG-3′ and

reverse primer,  5′-CACATTGGGGGTAGGAACAC-3′;

U6 small nuclear RNA forward primer, 5′-CTCGCTTCGGCAGCACA-3′ and

reverse primer, 5′-AACGCTTCACGAATTTGCGT-3′.

The experiments were repeated thrice independently.

### Western Blot Analysis

Cells were harvested and lysed with radioimmunoprecipitation assay (RIPA) buffer on ice for the isolation of total proteins. Cells were centrifuged at 10,000 × *g* for 10 min and the supernatants were carefully collected and used as total protein after detection of protein concentration using bicinchoninic acid (BCA) kit (Thermo Fisher Scientific). The cell lysate was separated using electrophoresis and the separated protein bands were transferred onto polyvinylidene difluoride (PVDF) membranes, followed by blocked using 5% fat-free milk for 1 h. Then, the membranes were incubated with primary antibodies at 4°C overnight. The next day, membranes were then treated with a fluorescent second antibody for 1 h and visualized with a chemiluminescence system (Millipore). The experiments were repeated thrice.

### Dual-Luciferase Reporter Assay

The cDNA of the wild-type or mutated Smad3 containing the binding site of miR-16-5p at the 3′-UTR were synthesized by GenScript (Nanjing, Jiangsu, China) and integrated into pmirGLO luciferase reporter vector (Promega, Madison, WI, USA). MG63 and HOS cells were seeded in 96-well plates and incubated for 24 h. Cells were transfected with the wild-type or mutated luciferase reporter vector along with miR-16-5p mimic, miR-NC, miR-16-5p inhibitor, or NC inhibitor for 48 h. Luciferase activity was detected using a Dual-Luciferase Assay Kit (Promega, CA, USA).

### Wound Healing Assay

A total of 1 × 10^5^ MG63 cells/well were seeded in six-well plates and allowed to attach. Cells were transfected with miR-16-5p mimic or miR-NC and cultured until 90% confluence. A scratch was produced with a pipette tip in the middle of every well and the healing rate was observed at 24 and 48 h. The experiments were repeated thrice.

### Transwell Invasion Assays

We used 24-well Transwell inserts (Corning Costar, Cambridge, MA, USA) to investigate the invasive capability of osteosarcoma cells. A total of 5 × 10^4^ MG63 cells transfected with miR-16-5p mimic, miR-NC, miR-16-5p inhibitor, or NC inhibitor in 200 μl of serum-free media were seeded onto the upper chamber of Transwell inserts and 500 μl of media containing 10% FBS used as a chemoattractant were added into the lower chambers. After 48 h, the cells in the upper chamber were removed with a swab and the inferior surface of the membrane was stained with 0.1% crystal violet after treatment with 4% paraformaldehyde. Positively stained cells were counted in five fields per well under a microscope. Experiments were repeated thrice.

### Immunohistochemistry Assays

Human osteosarcoma samples or adjacent tissues were fixed with formalin, embedded into paraffin, and sectioned as 5 μm slices. After deparaffinization and dehydration in graded ethanol solutions, tissue sections were incubated in 3% hydrogen peroxide solution for blocking endogenous peroxidases. The tissues were treated with a primary antibody against Smad3 at 4°C overnight. Following incubation, the samples were treated with a horseradish peroxidase (HRP)-conjugated secondary antibody at room temperature for 2 h. The sections were washed, counterstained with diaminobenzidine (DAB), and visualized under a microscope.

### Terminal Deoxynucleotidyl Transferase-Mediated Deoxyuridine Triphosphate-Biotin Nick End Labeling Staining

The apoptotic level of tumor cells in tumor tissue were evaluated by terminal deoxynucleotidyl transferase (TdT)-mediated deoxyuridine triphosphate (dUTP)-biotin nick end labeling (TUNEL) using an In Situ Cell Death Detection Kit (Roche, Basel, Switzerland) according to the manufacturer’s instructions. The tumor tissue sections were deparaffinized and rehydrated in an alcohol gradient. After washing with phosphate buffered saline (PBS), the sections were stained with the TUNEL reaction mixture, followed counterstaining with 4′,6-diamidino-2-phenylindole (DAPI) (1 μg/ml). The results were visualized by green fluorescence was visualized in cells under a fluorescence microscope (Olympus, Beijing, China).

### Statistical Analysis

All data were recorded as mean ± standard deviation (SD) from at least three independent experiments. Student’s *t*-test or chi-square test was used to analyze results using SPSS 21.0 software (Chicago, IL, USA). A value of p < 0.05 indicated significant difference.

## Results

### miR-16-5p Expression Is Downregulated in Osteosarcoma

**Figure 1:**
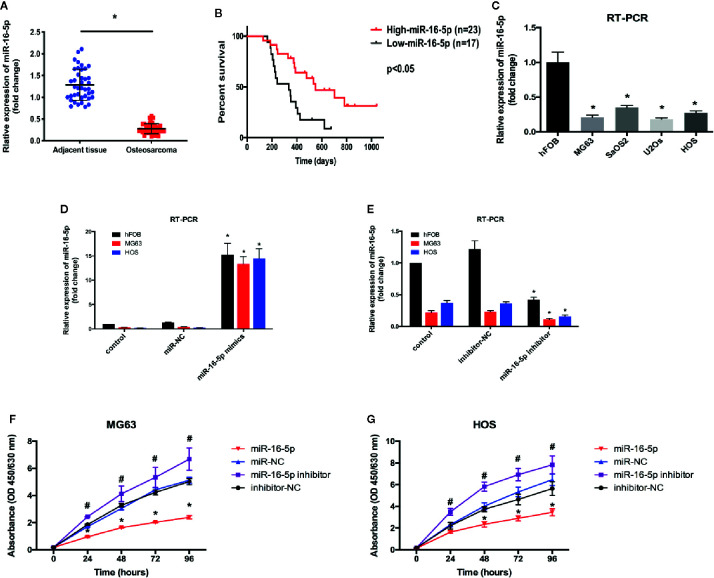
The expression level of miR-16-5p in osteosarcoma and normal cells. **(A**, **C)** miR-16-5ps in human osteosarcoma and adjacent tissue samples and cell lines were detected by real-time PCR (RT-PCR). **(B)** Kaplan-Meier survival rates for overall survival (OS) patients with high (n=23) and low (n=17) miR-16-5p expression. **(D**, **E)** miR-16-5p in cells transfected with mimics, inhibitor, and negative control were detected by RT-PCR. **(F**, **G)** the cell proliferation was evaluated by CCK-8. *indicates p < 0.05.

We evaluated the expression level of miR-16-5p in osteosarcoma and adjacent tissues using RT-PCR and found it to be significantly downregulated in osteosarcoma tissues as compared with adjacent tissues (**Figure 1A**). Retrospective Kaplan-Meier analysis of clinical samples revealed that the patients with high expression of miR-16-5p showed significantly longer survival than those with low expression of miR-16-5p, as analyzed with log-rank test (p < 0.01, **Figure 1B**). These results suggest that miR-16-5p expression inversely correlated with overall survival of patients. We evaluated the expression of miR-16-5p in osteosarcoma cell lines and a human osteoblast cell line. Consistent with the observations reported in tissues, the level of miR-16-5p in hFOB1.19 cells was remarkable higher than that in MG63, SaOS2, U2OS, and HOS cells (**Figure 1C**). To evaluate the effects of transfection, miR-16-5p expression level was detected in MG63 and HOS cells transfected with miR-16-5p mimic, miR-NC, miR-16-5p inhibitor, and NC inhibitor with RT-PCR. As a result, miR-16-5p was successfully overexpressed and repressed after transfection with mimic and inhibitor, respectively (**Figures 1D, E**). The proliferation of the cells transfected with miR-16-5p mimic, miR-NC, miR-16-5p inhibitor, or NC inhibitor was measured with CCK-8. Overexpression of miR-16-5p following transfection of miR-16-5p mimic resulted in the suppression of MG63 and HOS cell proliferation. On the contrary, the inhibition of miR-16-5p expression following transfection with miR-16-5p inhibitor greatly promoted the proliferation of osteosarcoma cells (**Figures 1F, G**).

### miR-16-5p Inhibits the Migration and Invasion of Osteosarcoma Cells

**Figure 2:**
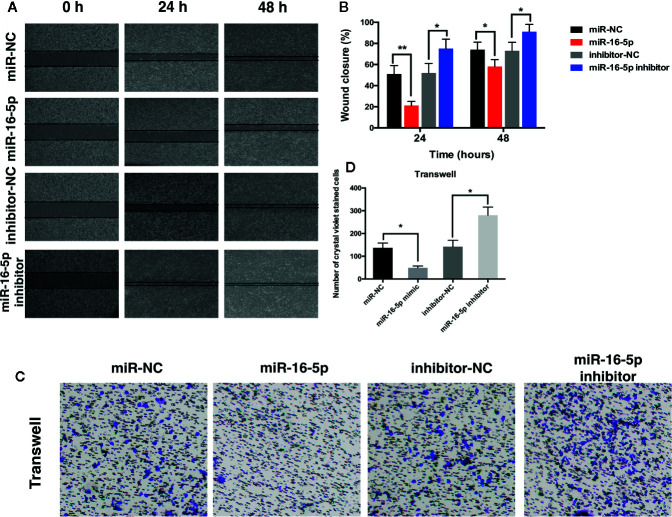
Migration and invasion of osteosarcoma cells were inhibited by miR-16-5p. **(A**, **B)** wound-healing assays were performed using MG63 cells transfected with miR-16-5p mimics, miR-NC, miR-16-5p inhibitor, or inhibitor-NC for evaluating cells migration, and percentages of closure at 24 and 48 h were recorded. **(C**, **D)** Transwell assays and crystal violet staining were performed to detect the invasive capability of MG63 cells transfected with miR-NC, miR-16-5p mimics, miR-16-5p inhibitor, and inhibitor-NC, and positive stained cells was calculated. *indicates p < 0.05.

Wound healing assay was performed to investigate the effect of miR-16-5p expression on the migration of MG63 cells transfected with miR-NC, miR-16-5p mimic, miR-16-5p inhibitor, or NC inhibitor. After 24 and 48 h of incubation, the percentage of wound closure was evaluated. As a result, the wound in the group transfected with miR-16-5p mimic was remarkably wider than that in the control group transfected with miR-NC (p < 0.05). The width of wound in miR-16-5p inhibitor-transfected cells was significantly smaller than that reported in control cells transfected with NC inhibitor (p < 0.05, **Figures 2A, B**). Thus, the upregulation of miR-16-5p expression inhibited the migration of osteosarcoma cells. Transwell invasion assay showed that the number of cells stained positive with crystal violet was lower in the group transfected with miR-16-5p (49 ± 8) than in the control group transfected with miR-NC (137 ± 21) (p < 0.05, **Figures 2C, D**). However, the number of positively stained cells in the group transfected with miR-16-5p inhibitor (281 ± 36) was significantly higher than that in the control group transfected with NC inhibitor (142 ± 28) (p < 0.05, **Figures 2C, D**). Therefore, the overexpression of miR-16-5p significantly decreased the invasion of osteosarcoma cells. Overall, miR-16-5p expression inhibited the migration and invasion of osteosarcoma cells and promote the apoptosis of osteosarcoma cells *in vitro*.

### miR-16-5p Targets Smad3 in Osteosarcoma

**Figure 3:**
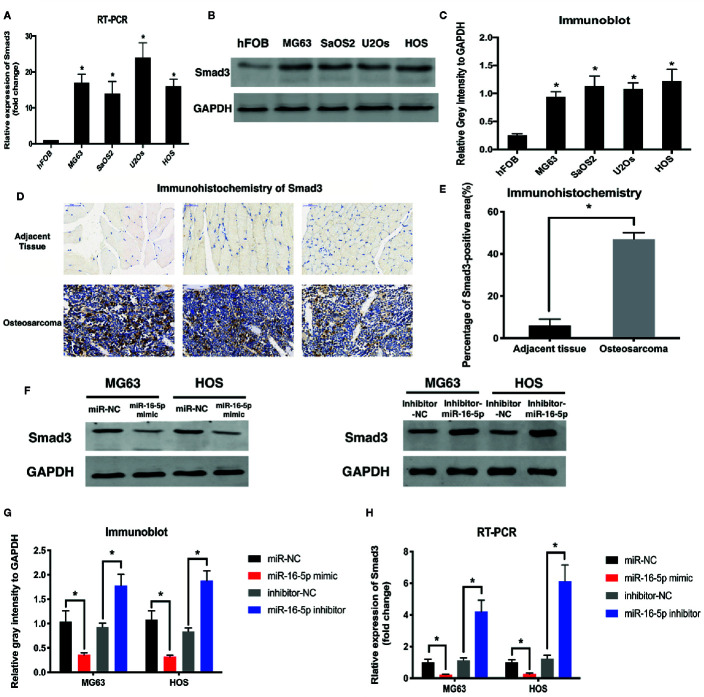
Smad3 was regulated by miR-16-5p in osteosarcoma. **(A)** Real-time PCR (RT-PCR) and **(B**, **C)** immunoblot were performed to detect the expression of Smad3 in human osteoblasts and osteosarcoma cell lines. **(D**, **E)** Smad3 was analyzed by immunohistochemistry assays in human osteosarcoma and adjacent tissue. Smad3 in MG63 and HOS cells transfected with miR-16-5p mimics or inhibitor were detected by immunoblot **(F**, **G)** and RT-PCR **(H)**. *indicates p < 0.05.

Results of RT-PCR analysis showed that the mRNA expression of Smad3 was upregulated in MG63, SaOS2, U2OS, and HOS cells at different levels as compared with that in hFOB1.19 cells (**Figure 3A**). Immunoblot assay revealed the increase in the expression of Smad3 in osteosarcoma cell lines (**Figures 3B, C**). The expression of Smad3 in human osteosarcoma specimens was detected with immunohistochemistry. Smad3 was overexpressed in the osteosarcoma tissue as compared with the adjacent tissue (**Figures 3D, E**). We evaluated Smad3 expression in osteosarcoma cells transfected with miR-16-5p mimic, miR-NC, miR-16-5p inhibitor, or NC inhibitor with western blotting. The overexpression of miR-16-5p resulted in the suppression of Smad3 expression in osteosarcoma cells and this suppressive effect was attenuated following downregulation of miR-16-5p expression (**Figures 3F, G**). The results of RT-PCR showed that the mRNA expression of Smad3 decreased in MG63 and HOS cells transfected with miR-16-5p mimic as compared with that in control cells. The transfection of miR-16-5p inhibitor remarkably suppressed the expression of Smad3 in MG63 and HOS cells (**Figure 3H**). Therefore, miR-16-5p targeted *Smad3* mRNA in MG63 and HOS cells, and this effect was abrogated following miR-16-5p inhibition.

### Overexpression of miR-16-5p Enhances the Susceptibility of Osteosarcoma Cells to Cisplatin

**Figure 4:**
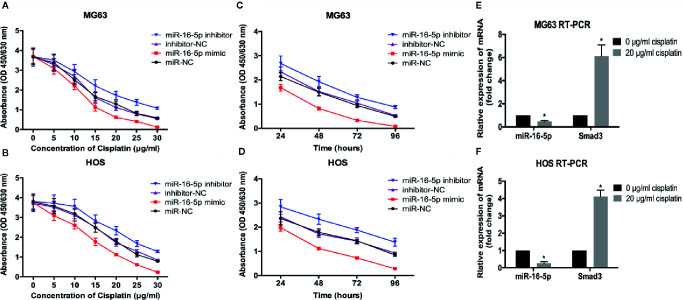
Therapeutic efficacy of cisplatin in osteosarcoma cells was enhanced by over-expression of miR-16-5p. the cell viabilities of MG63 and HOS cells treated with varying concentrations of cisplatin **(A**, **B)** for 48 h and with 20 μg/ml for different times **(C**, **D)**. **(E**, **F)** miR-16-5p and Smad3 were detected by real-time PCR (RT-PCR) in cells incubated with or without 20 μg/ml cisplatin. *indicates p < 0.05.

To investigate the effect of miR-16-5p on the chemoresistance of osteosarcoma, MG63 and HOS cells transfected with miR-16-5p mimic, miR-NC, miR-16-5p inhibitor, and NC inhibitor were cultured in the presence of different concentrations of cisplatin for 48 h. Cell viability was detected with CCK-8. As a result, the increase in miR-16-5p expression significantly enhanced the antitumor effects of cisplatin as compared with the control cells, while the inhibition of miR-16-5p expression promoted the resistance of MG63 and HOS cells to cisplatin treatment (**Figures 4A, B**) at 24, 48, 72, and 96 h of incubation (**Figures 4C, D**). Furthermore, osteosarcoma cells were incubated with cisplatin for 48 h, and the expression of *Smad3* mRNA and miR-16-5p was detected with RT-PCR. As a result, we found that the expression of miR-16-5p was downregulated, while that of *Smad3* was upregulated.

### miR-16-5p Directly Targets Smad3

**Figure 5:**
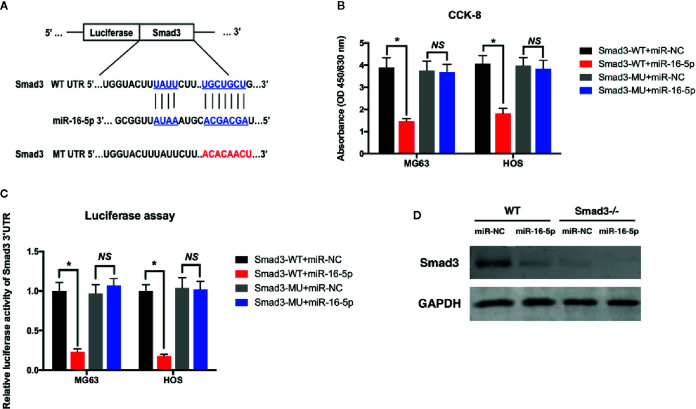
miR-16-5p inhibits osteosarcoma cells directly targeting at Smad3. **(A)** A mutated Smad3 expression vector was constructed and transfected into MG63 and HOS cells. **(B)** the cell proliferations of wild type or mutated MG63 cells transfected with miR-NC or miR-16-5p mimics were detected using CCK-8 assays. **(C)** Transcription activity of Smad3 in wild type and mutated MG63 and HOS cells were measured by dual-luciferase assays. **(D)** Smad3 was detected in wild type and Smad3-/- cells transfected with miR-NC or miR-16-5p mimics. *indicates p < 0.05.

To evaluate the effect of Smad3 targeting of miR-16-5p on the growth and chemoresistance of osteosarcoma, the vector expressing the mutated 3′-UTR of Smad3 was transfected into MG63 and HOS cells along with miR-16-5p mimic or inhibitor (**Figure 5A**). According to the results of cell viability assay, the proliferation of the cells expressing wild-type Smad3 and miR-16-5p mimic was remarkably inhibited as compared with that of the control cells (p < 0.05). However, the inhibitory effect of miR-16-5p was completely attenuated following mutation of Smad3 (p > 0.05, **Figure 5B**). The transcriptional activity of Smad3 in MG63 and HOS cells was measured with a dual-luciferase reporter assay. The luciferase activity was significantly lower in the cells transfected with the wild-type Smad3 and miR-16-5p mimic than in those transfected with the wild-type Smad3 and miR-NC (MG63: 1 *versus* 0.23 ± 0.04, HOS: 1 *versus* 0.18 ± 0.02, p < 0.05). No reduction in luciferase activity was observed in the cells expressing the mutated Smad3 and miR-16-5p mimic (MG63: 0.97 ± 0.11 *versus* 1.07 ± 0.08; HOS: 1.04 ± 0.13 *versus* 1.02 ± 0.10, p > 0.05). Therefore, overexpression of miR-16-5p resulted in a decrease in the transcriptional activity of Smad3 in MG63 and HOS cells, while this effect was attenuated in the cells expressing mutated Smad3 (**Figure 5C**). We investigated whether miR-16-5p decreases the expression of Smad3 protein by silencing the expression of Smad3 with the transfection of small-interfering RNA (siRNA)-Smad3. As a result, Smad3 expression was inhibited by miR-16-5p transfection in wild-type cells. The inhibitory effect of miR-16-5p on Smad3 expression was associated with the effect of siRNA-Smad3 (**Figure 5D**).

## Discussion

Many studies have evaluated the functions of miRNAs on the physiological and pathological behaviors of cells, including osteosarcoma cells (Shen et al., 2018; Xie et al., 2018). miRNAs not only regulate the carcinogenesis and metastasis of osteosarcoma cells but also influence their sensitivity to chemotherapy. miR-199a-3p was demonstrated to regulate drug resistance by targeting adenylate kinase 4 (AK4) (Lei et al., 2018). In addition, miR-140 expression was shown to overcome the chemoresistance of both osteosarcoma and colon cancer cells (Song et al., 2009). At present, very few studies have evaluated the function of miR-16-5p, and miR-16-5p may serve as a biomarker of early-stage rheumatoid arthritis, (Dunaeva et al., 2018) and gastric cancer progression (Zhang et al., 2015). Differentiation of myoblasts was regulated by miR-16-5p expression through SESN1 targeting, thereby affecting cell proliferation and apoptosis (Cai et al., 2018). However, the role of miR-16-5p in osteosarcoma is yet questionable.

In the present study, we detected the expression of miR-16-5p in human osteosarcoma and adjacent tissues and cell lines. miR-16-5p expression was significantly reduced in both osteosarcoma samples and multiple cell lines as compared with adjacent counterparts (**Figure 1**). To investigate the function of miR-16-5p, the mimic and inhibitor of miR-16-5p were designed and synthesized and their effects were verified by RT-PCR. Overexpression of miR-16-5p resulted in the inhibition of the proliferation, migration, and invasion of osteosarcoma cells and this effect was attenuated following transfection with miR-16-5p inhibitor (**Figure 2**). miR-16-5p is known to inhibit the growth and metastasis of chordoma by targeting the expression of Smad3 (Zhang et al., 2018). Furthermore, melatonin was shown to suppress the proliferation of gastric cancer cells by promoting the expression of miR-16-5p, which targeted Smad3 (Zhu et al., 2018). Therefore, we hypothesized that miR-16-5p downregulates Smad3 expression in osteosarcoma cells, thereby inhibiting their proliferation and enhancing their sensitivity to chemotherapy. We detected the expression of Smad3 in multiple human osteosarcoma cell lines and a human osteoblast cell line. The results of RT-PCR and immunoblot analysis demonstrated that Samd3 expression increased in osteosarcoma cells as compared with normal cells, consistent with the observations reported in human osteosarcoma samples (**Figure 3**). miR-16-5p mimic and inhibitor were separately transfected into MG63 and HOS cells. Overexpression of miR-16-5p significantly reduced Smad3 expression, which may be restored following transfection of cells with miR-16-5p inhibitor. Taken together, miR-16-5p suppressed the proliferation, migration, and invasion of osteosarcoma cells by regulating the expression of Smad3. Qu, et al. reported that the overexpression of miR-16-5p in breast cancer cells might inhibit cell proliferation and invasion and induce apoptosis *via* vascular endothelial growth factor A targeting (Qu et al., 2017).

We investigated the effect of miR-16-5p expression on the sensitivity of osteosarcoma cells to cisplatin. The therapeutic effects of cisplatin in the cells transfected with miR-16-5p mimic was obviously enhanced in a dose- and time-dependent manner. Inhibition of miR-16-5p expression resulted in a significant manifestation in the resistance of these cells to cisplatin. Incubation of osteosarcoma cells with 20 μg/ml of cisplatin for 48 h resulted in the reduction in the level of miR-16-5p with a simultaneous increase in Smad3 expression (**Figure 4**). Therefore, miR-16-5p increased the chemotherapeutic sensitivity of osteosarcoma cells to cisplatin. It was revealed that TGF-β can up-regulate the expression of HSP27 and cisplatin resistance in human lung cancer cell through blocking the cisplatin-induced apoptosis and cell death, which characterized as the increasing of cell viability and decreasing of PARP and caspase3 cleavage in the cisplatin-treated cell. Knockdown of SMAD3 attenuated the TGF-β-induced HSP27 expression and enhanced the chemosensitivity of human lung cancer to cisplatin (Huang et al., 2017). In our study, miR-16-5p can down-regulate Smad3 to promote the anti-cancer effect of cisplatin on osteosarcoma. Moreover, previous research demonstrated that miR-149-5p increased the chemosensitivity of cisplatin on oral squamous cell carcinoma *via* inhibiting TGF-β2, p-Smad2, and p-Smad3 (Luo et al., 2019). Silencing TGF-β or smad3 in A549/TAX (paclitaxel resistant) cells decreased the expression of cathepsin L and enhanced their sensitivity to paclitaxel (Zhao et al., 2019).

In addition, an expression vector carrying mutated or wild-type Smad3 was constructed and transfected into osteosarcoma cells. Overexpression of miR-16-5p greatly suppressed the proliferation of osteosarcoma cells. However, this suppressive effect of miR-16-5p on the proliferation of osteosarcoma cells was attenuated in the cells expressing mutated Smad3, wherein miR-16-5p was unable to recognize and bind to the mutated 3′-UTR of Smad3. Therefore, the transcription activity of Smad3 was suppressed by miR-16-5p in the cells expressing wild-type Smad3 but not mutated Smad3 (**Figure 5C**). The translation of Smad3 was also inhibited following overexpression of miR-16-5p (**Figure 5D**). We investigated the inhibitory effect of miR-16-5p on the expression of Smad3 by silencing Smad3 expression through siRNA-Smad3 transfection. As a result, the inhibitory effect of miR-16-5p on Smad3 expression was close to the effect observed with siRNA-Smad3 (**Figure 5D**). Finally, the effect of miR-16-5p on osteosarcoma *in vivo* was also investigated with xenograft nude model. Consequently, the tumor volume in miR-NC and miR-16-5p injected groups were measured and it was demonstrated that the miR-16-5p remarkably inhibited MG63 cells proliferation and induced their apoptosis *in vivo*. Further, the Smad3 in miR-16-5p injected group were also down-regulated in line with *ex vivo* results.

Taken together, we demonstrate that miR-16-5p expression is downregulated in osteosarcoma and that overexpression of miR-16-5p inhibits the proliferation, migration, and invasion of osteosarcoma cells by targeting Smad3. Moreover, miR-16-5p overexpression enhances the chemotherapeutic sensitivity of osteosarcoma cells to cisplatin.

## Data Availability Statement

All datasets generated for this study are included in the article/**Supplementary Material**.

## Ethics Statement

The studies involving human participants were reviewed and approved by Institutional Review Board of Tongren Hospital (No. 2019-015). The patients provided their written informed consent to participate in this study. The informed consent in accordance with the Declaration of Helsinki were signed and obtained from all donors.​

## Author Contributions

WX and ZG initially designed the whole study. ZG, ZL, and XZ performed cellular experiments including cell culture, RT-PCR, western blot assay. Also, ZG, ZL, RH, and YX performed animal experiments. ZL, YX and WX analyzed all data. Eventually, ZG wrote and revised the manuscript under the instructions of YX and WX. All authors contributed to the article and approved the submitted version.

## Funding

The study was supported by Excellent Young Medical Talents Training Plan of Shanghai Health Planning Commission (2018YQ46) and K. C. WONG Education Foundation (Hong Kong).

## Conflict of Interest

The authors declare that the research was conducted in the absence of any commercial or financial relationships that could be construed as a potential conflict of interest.
